# Risk factors for bacteremia in children with febrile neutropenia

**DOI:** 10.3906/sag-1901-90

**Published:** 2019-08-08

**Authors:** Soner Sertan KARA, Hasan TEZER, Meltem POLAT, Burcu Ceylan CURA YAYLA, Tuğba BEDİR DEMİRDAĞ, Arzu OKUR, Ali FETTAH, Saliha KANIK YÜKSEK, Anıl TAPISIZ, Zühre KAYA, Namık ÖZBEK, İdil YENİCESU, Neşe YARALI, Ülker KOÇAK

**Affiliations:** 1 Department of Pediatric Infectious Diseases, Faculty of Medicine, Gazi University, Ankara Turkey; 2 Department of Pediatric Oncology, Faculty of Medicine, Gazi University, Ankara Turkey; 3 Department of Pediatric Infectious Diseases, Ankara Hematology Oncology Children’s Training and Research Hospital, Ankara Turkey; 4 Department of Pediatric Hematology-Oncology, Ankara Hematology Oncology Children’s Training and Research Hospital, Ankara Turkey; 5 Department of Pediatric Hematology, Faculty of Medicine, Gazi University, Ankara Turkey

**Keywords:** Bacteremia, children, febrile neutropenia, risk factor

## Abstract

**Background/aim:**

Bacteremia remains an important cause of morbidity and mortality during febrile neutropenia (FN) episodes. We aimed to define the risk factors for bacteremia in febrile neutropenic children with hemato-oncological malignancies.

**Materials and methods:**

The records of 150 patients aged ≤18 years who developed FN in hematology and oncology clinics were retrospectively evaluated. Patients with bacteremia were compared to patients with negative blood cultures.

**Results:**

The mean age of the patients was 7.5 ± 4.8 years. Leukemia was more prevalent than solid tumors (61.3% vs. 38.7%). Bacteremia was present in 23.3% of the patients. Coagulase-negative staphylococci were the most frequently isolated microorganism. Leukopenia, severe neutropenia, positive peripheral blood and central line cultures during the previous 3 months, presence of a central line, previous FN episode(s), hypotension, tachycardia, and tachypnea were found to be risk factors for bacteremia. Positive central line cultures during the previous 3 months and presence of previous FN episode(s) were shown to increase bacteremia risk by 2.4-fold and 2.5-fold, respectively.

**Conclusion:**

Presence of a bacterial growth in central line cultures during the previous 3 months and presence of any previous FN episode(s) were shown to increase bacteremia risk by 2.4-fold and 2.5-fold, respectively. These factors can predict bacteremia in children with FN.

## 1. Introduction

Febrile neutropenia (FN) is a leading cause of infectious mortality for patients receiving cytotoxic chemotherapies. Approximately one-third of children with cancer treatment or hematopoietic stem cell transplantation experience FN during the neutropenic period [1]. During these episodes, bacteremia remains one of the most important causes of morbidity and mortality due to serious complications. 

Neutropenia significantly changes the inflammatory response of the host, and it is therefore difficult to identify infections. Documented infectious etiology is encountered in only 20%–30% of febrile neutropenic patients [2]. Blood culture remains the mainstay for the diagnosis of bacteremia. Bacterial isolation rates using the conventional technique vary between 25%–80% depending on the clinical situation of children with FN [3]. Thus, defining the risk factors is crucial for rapid and prompt diagnosis and eventual appropriate treatment in order to obviate high rates of morbidity and mortality. 

Although some risk stratifications have been suggested to identify low-risk episodes of FN in adults, fever is sometimes the only sign of severe and life-threatening infections in neutropenic children with cancer [4]. There is no validated risk scoring system for children with FN to determine the high-risk groups for development of bacteremia, but some clinical parameters such as diagnosis of leukemia [5], induction type chemotherapy [6], neutropenia for longer than 7 days [5,7], body temperature of over 39 °C at the time of FN diagnosis [8], presence of chills or hypotension and the need for resuscitation [9], presence of central venous catheters (CVCs) [10], and laboratory parameters such as increased C-reactive protein, procalcitonin and interleukin levels [5,11,12], and absolute neutrophil counts of ≤200/mm3 have been proposed previously in children and adults. 

In this study, we aimed to investigate the presence of risk factors for bacteremia in febrile neutropenic children with hemato-oncological malignancies. 

## 2. Materials and methods

### 2.1. Study design and patient population

The records of 150 pediatric patients aged 18 years and younger with hemato-oncological malignancies who developed FN in the hematology and oncology clinics of the Gazi University Medical Faculty and the Ankara Hematology Oncology Children’s Training and Research Hospital from 1 January 2014 to 31 December 2015 were retrospectively evaluated. The data of patients with hematopoietic stem cell transplantation, patients who did not fulfill the criteria for FN, and patients with fungemia were excluded. Only the first FN episode of each patient during this period was included and subsequent episodes were not included. 

### 2.2. Data collection

The medical records of the enrolled patients were collected, including clinical characteristics and laboratory findings on the first day of FN episode: age, sex, primary diagnosis, presence of previous FN episode(s), duration since the last chemotherapy administration, type of last chemotherapy, duration and severity of neutropenia, duration and maximum value of fever, duration of hospitalization, antibiotic usage (type and duration), presence of hypotension, tachycardia, tachypnea, focal infection, indwelling catheter (central line [central venous catheter or implantable port] and urinary catheter), and laboratory parameters (white blood cell count [WBC], absolute neutrophil count [ANC], hemoglobin, platelet count, C-reactive protein [CRP], erythrocyte sedimentation rate [ESH], abnormality in liver and renal functions, electrolyte imbalance, urine analysis and growth in peripheral blood and central venous catheter [CVC]/implantable port [IP], urine cultures, and cultures from other sites, such as skin lesions, cerebrospinal fluid [CSF], throat, or stools [if present]). Also, positivity in peripheral blood and/or CVC/IP cultures during the previous 3 months was recorded. The study was approved by the local ethics committee (date: 05/17/2016; number: 2016-10-65). 

### 2.3. Definitions 

Febrile neutropenia is defined as a fever, which is a single measurement of ≥38.3 °C or a temperature of ≥38 °C lasting for over 1 h, plus an ANC of <500/mm3 or expected to decrease <500/mm3 within 48 h [13]. The maximum value of fever at the time of diagnosis was the highest documented value of the body temperature measured in the emergency department/clinic until FN diagnosis was established. Hypotension was defined as systolic blood pressure below the fifth percentile for age and sex, or the need for vasopressor support. Tachycardia was defined as mean heart rate >2 standard deviations (SDs) above normal for age, not resulting from external stimulus, chronic drugs, or painful stimuli. Tachypnea was defined as mean respiratory rate >2 SDs above normal for age. Duration since last chemotherapy was the sum of days passed since the first day of the last chemotherapy period. 

Two sets of blood culture from two separate veins were drawn from each patient at the time of FN diagnosis. If present, blood samples were also obtained from each lumen of any central line. Collected blood samples were placed into BACTEC culture vials and cultures were determined to be positive by an automated continuous monitoring system (BACTEC 9240; Becton Dickson, Sparks, MD, USA). Bacteremia was defined as the isolation of a significant pathogen from one or more blood cultures that was not a contaminant when the patient had clinical symptoms and signs of infection. After discriminating between true bacteremia and contamination, all cases fulfilling the CDC-NHSN definition of clinically significant bloodstream infection were included [14]. Coagulase-negative staphylococci (CNS) are the most frequent contaminants of blood cultures. To distinguish true infection from contamination, the presence of systemic manifestations of infection, such as tachycardia, tachypnea, and hypotension in addition to fever was noted, or at least two separate positive blood culture results repeated 1–2 days apart from each other were considered as positive to render the diagno­sis of true infection relatively secure. 

### 2.4. Statistical analysis

Statistical analyses were performed using SPSS 18 (SPSS Inc., Chicago, IL, USA). The Shapiro–Wilk test was used to investigate whether the continuous variables were normally distributed. Descriptive analyses were presented using means and standard deviations and medians and minimum–maximum for normally and nonnormally distributed variables, respectively. If normally distributed, an independent t samples test was used, and if not normally distributed, the Mann–Whitney U test was used to compare two independent groups. Chi-square or Fischer exact tests were used for comparisons between categorical variables. The variables that turned out to be significant for bacteremia after univariate analysis (white blood cell count, presence of central line, severity of neutropenia at admission, presence of previous FN episode(s), positivity in peripheral blood, and CVC/IP cultures during previous 3 months) were included in regression analyses (enter model) to explore independent risk factors for bacteremia. P < 0.05 was considered to show a statistically significant result. 

## 3. Results

One hundred and fifty patients were enrolled in the study. The mean age of the children was 7.5 ± 4.8 years (range: 3.6 months to 18 years) and the male/female ratio was 1.5 (92 [61.3%] / 58 [38.7%]). Leukemia was more prevalent than solid tumors (61.3% vs. 38.7%). Other clinical characteristics of the study population are shown in Table 1.****

**Table 1:** Clinical characteristics of the study population (n = 150).

	n (%)
Primary diagnosisa Leukemiab Solid tumor	92 (61.3%)58 (38.7%)
Presence of previous FN episode(s)	104 (69.3%)
Duration since the last chemotherapy administered (days) [median (min–max)]	8 (0–50)
Type of the last chemotherapyInductionConsolidationMaintenance	93 (62%)41 (27.3%)16 (10.7%)
Duration of neutropenia<7 days≥7 days	93 (62%)57 (38%)
Severity of neutropenia at admission <100/mm3100–500/mm3	102 (68%)48 (32%)
Duration of fever at the time of blood drawn First 12 h12–24 h>48 h	138 (92%)11 (7.3%)1 (0.7%)
Duration of hospitalization at admissionHospitalized for ≥48 hComing from home	88 (58.7%)62 (41.3%)
Prophylactic or treatment antibiotic usage at the time of diagnosis	85 (56.7%)
Tachycardia	6 (4.0%)
Hypotension	6 (4.0%)
Tachypnea	4 (2.6%)
Focal infection/inflammation URTIOral mucositisGastroenteritis PneumoniaAnal abscessCellulitis UTIDental carriesInfection at catheter entrance siteTyphlitis Zona infection	98 (65.3%)50 (33.3%)41 (27.3%)21 (14.0%)7 (4.7%)6 (4.0%)5 (3.3%)4 (2.7%)2 (1.3%)2 (1.3%)2 (1.3%)1 (0.6%)
Presence of central lineCentral venous catheterPort device	107 (71.3%)81 (54.0%)26 (17.3%)
Presence of urinary catheter	9 (6.0%)
Max value of fever at the time of diagnosis (°C) (mean ± SD)	38.4 ± 0.3

SD, standard deviation; FN, febrile neutropenia; min, minimum; max, maximum. a Acute lymphoblastic leukemia (ALL), 64 (42.7%); acute myeloblastic leukemia (AML), 20 (13.3%); juvenile myelomonocytic leukemia, 2 (1.3%); relapse ALL, 4 (2.7%); relapse AML, 1 (0.7%). bNon-Hodgkin lymphoma/Burkitt lymphoma, 12 (8%); neuroblastoma, 12 (8%); Ewing sarcoma, 10 (6.7%); rhabdomyosarcoma, 4 (2.7%); retinoblastoma, 4 (2.7%); medulloblastoma, 3 (2%); Hodgkin lymphoma, 2 (1.3%); germ cell tumor, 2 (1.3%); yolk sac tumor, 2 (1.3%); rhabdoid tumor, 2 (1.3%); glioblastoma multiforme, 1 (0.7%); oligodendroglioma, 1 (0.7%); synovial sarcoma, 1 (0.7%); primitive neuroectodermal tumor (PNET), 1 (0.7%); ependymoma, 1 (0.7%); astrocytoma, 1 (0.7%); SD, standard deviation; URTI, upper respiratory infection; UTI, urinary tract infection.

Laboratory characteristics of the study population are shown in Table 2. Thirty-five (23.3%) patients were shown to have blood culture positivity. Eleven (31.4%) CNS (penicillin-sensitive, 3 [8.5%]; oxacillin-sensitive, 3 [8.5%]; and oxacillin-resistant, 5 [14.2%]), 6 (17.1%) *Escherichia coli*, 5 (14.2%) *Klebsiella pneumoniae*, 4 (11.4%) *Staphylococcus aureus* (penicillin-sensitive, 3 [8.5%]; oxacillin-resistant, 1 [2.8%]), 4 (11.4%) *Stenotrophomonas maltophilia*, 2 (5.7%) *Enterococcus faecalis *(vancomycin-sensitive), 1 (2.8%) alpha-hemolytic streptococcus, 1 (2.8%) *Pseudomonas aeruginosa*, and 1 (2.8%) *Pseudomonas oryzihabitans* growths were seen in blood cultures. No mixed infections were observed. Eleven (7.3%) patients who also had peripheral blood culture positivity had growth in the CVC/IP culture. The pathogens were as follows: CNS, 5 (45.4%); *Escherichia coli*, 3 (27.2%); *Klebsiella pneumoniae*, 2 (18.1%); and *Pseudomonas aeruginosa*, 1 (9.0%). No mixed infections were observed. Ten (6.7%) patients had urinary culture positivity; no patients had CSF or stool culture positivity. 

**Table 2:** Laboratory characteristics of the study population (n = 150).

Parameter	Value
WBC (/µL) [median (min–max)]	600 (0–26,500)
ANC (/µL) [median (min–max)]	40 (0–500)
Hemoglobin value (g/dL) (mean ± SD)	8.8 ± 1.5
Platelet count (/µL) (median [min–max])	38,550 (3200–559,000)
CRP level (0–5 mg/dL) [median (min–max)]	28.5 (1–438)
ESH (0–20 mm/h) (mean ± SD)	52.5 ± 32.6
Growth in peripheral blood culture, n (%)	35 (23.3%)
Growth in central line culture, n (%)	11 (7.3%)
Presence of urinary tract infection, n (%)	10 (6.7%)
Positivity in peripheral blood culture during previous 3 months, n (%)	37 (24.7%)
Positivity in central line culture during previous 3 months, n (%)*	31 (20.7%)
Abnormality in liver functions, n (%)	39 (26%)
Abnormality in renal functions, n (%)	4 (2.7%)
Electrolyte imbalance, n (%)	15 (10%)

WBC, white blood cell count; ANC, absolute neutrophil count; CRP, C-reactive protein; ESH, erythrocyte sedimentation rate; *, central venous catheter or implantable port.

Patients with peripheral blood culture positivity were compared with patients with negative blood cultures to demonstrate the risk factors for bacteremia during an FN episode (Table 3). White blood cell count, severity of neutropenia at admission, positivity in peripheral blood and central line cultures during the previous 3 months, presence of a central line, presence of previous FN episode(s), and presence of hypotension, tachycardia, and tachypnea were risk factors for bacteremia (P = 0.02, P = 0.01, P = 0.01, P < 0.0001, P = 0.03, P = 0.001, P < 0.0001, P < 0.0001, and P = 0.003, respectively). 

**Table 3:** Univariate analysis of risk factors of bacteremia in children with FN.

Parameter, n (%)	Bacteremia (+)	Bacteremia (–)	P-value
WBC (/µL) [median (min–max)]	420 (0–3150)	600 (80–26,500)	0.02
Severity of neutropenia at admission <100/mm3100–500/mm3	30 (85.7%)5 (14.2%)	72 (62.6%)43 (37.3%)	0.01
Positivity in peripheral blood culture during previous 3 months, n (%)	14 (40.0%)	23 (20.0%)	0.01
Positivity in central line culture during previous 3 months, n (%)*	15 (42.8%)	16 (13.9%)	<0.0001
Presence of central line*	30 (85.7%)	77 (67.0%)	0.03
Presence of previous FN episode(s)	32 (91.4%)	72 (62.6%)	0.001
Hypotension	6 (17.1%)	0 (0%)	<0.0001
Tachycardia	6 (17.1%)	0 (0%)	<0.0001
Tachypnea	4 (11.4%)	0 (0%)	0.003

FN, febrile neutropenia; WBC, white blood cell count; *, central venous catheter or implantable port.

In regression analyses, positivity in central line cultures during the previous 3 months (OR: 2.44; 95% CI: 1.16–5.12, P = 0.01) and presence of previous FN episode(s) (OR: 2.55; 95% CI: 1.27–5.15, P = 0.009) were shown to be independently associated with bacteremia in febrile neutropenic children, as shown in Table 4.

**Table 4:** Logistic regression analysis of risk factors for bacteremia in children with FN.

Independent risk factor	B	S.E.	Wald	Odds ratio	Confidence interval (95%)	P-value
Positivity in central line culture during previous 3 months, n (%)*	0.89	0.377	5.64	2.44	1.16–5.12	0.01
Presence of previous FN episode(s)	0.93	0.35	6.91	2.55	1.27–5.15	0.009

## 4. Discussion

In this study, the risk factors related to bacteremia in children with FN were investigated in two centers in a developing country. In general, although most episodes of FN are assumed to result from an infection, blood cultures were positive in less than 30% of febrile neutropenic episodes [15]. Similar to the literature, the present study had a rate of bacteremia of 23.3%.

Children with FN are a heterogeneous group with a varying risk of severe infection or medical complications. Management of infectious complications in these patients requires the definition and extinguishment of the risk factors. Knowledge regarding the previous history of FN and the risk of subsequent febrile episodes might be useful at the beginning of any chemotherapy-induced granulocytopenic period in order to decide on prophylactic or interventional strategies [16]. Although there are conflicting reports about this association in the literature, in this study, 69.3% of the patients had experienced at least one FN episode previously, and the presence of a history of FN episode(s) was shown to increase the risk of bacteremia in febrile neutropenic children by approximately 2.5 times for latter episodes [16–18]. Additionally, it was previously concluded that more than three previous FN episodes were related to adverse outcomes such as mortality, invasive infections, and hemodynamic instability in febrile neutropenic patients [19].

The results of this study revealed that positivity in central line cultures during the previous 3 months increased the risk of bacteremia by approximately 2.4 times. Having a history of bacteremia during the last 3 months was associated with bacteremia. In the literature, any previously documented infection in the past 6 months was shown to be a risk factor in pediatric FN for serious medical complications, including a microbiologically defined infection [20]. Catheter-associated blood stream infections are frequently seen during cancer treatment. Recurrent bacteremia in children with FN has been documented mostly when infected catheters could not be removed because of catheter salvage attempts [21]. Pathogens form a biofilm layer and hide from the killing effect of antibiotics. Additionally, any previous bacteremia due to other pathogens may lead to subsequent bacteremia in the host with more resistant pathogens [22]. However, such an association was not obtained in this study. Rondinelli et al. [10] reported that central lines were a risk factor for severe infectious complications, including bacteremia in FN patients. In this study, most of the patients had a central line, of which the majority were CVCs. It was found that the presence of a central line was associated with bacteremia in children with FN. Similarly, Al Omar et al. [23] stated that positive blood cultures were significantly more frequent in episodes with a CVC compared with episodes with no CVC. Additionally, CVCs are also a reason for bloodstream infections by multidrug-resistant bacteria [24]. 

This study revealed that patients with hypotension, tachycardia, and tachypnea were more prone to have bacteremia. In addition to the instability of these vital signs, the presence of abnormal body temperature or leukocyte count, together with suspected/present source of infection, is defined as sepsis. It was shown that over 50% of patients with FN develop sepsis syndrome, 20%–30% develop severe sepsis, and 5%–10% develop septic shock [25]. The NICE guidelines noted that the presence of hypotension and tachypnea were high risk factors for septic complications [26]. Although Asturias et al. [27] found no relationship between bacteremia and the simultaneous presence of hypotension, other studies reported that the presence of hypotension together with prolonged capillary refill time, fever above 39 °C, and pneumonia was related to invasive bacterial infections and fatal outcomes in bacteremic episodes in children with FN [11,28]. Additionally, “sick” or unwell general clinical appearance on admission was noted as a clue for significant/proven bacterial infection in children with FN [29].

Infectious complications seen in the neutropenic host are a direct result of faulty defense; a clear relationship is present with neutropenia, which develops because of the extended use of high-dose chemotherapeutics. The degree, rapidity of onset, and duration of neutropenia directly affect the rate and severity of infections because of diminished phagocytic activity of neutrophils against bacteria [30]. Previous reports stated that ANC of <100/mm3 was shown to predict the risk of invasive bacterial infections in pediatric cancer patients with fever and neutropenia [26,29]. Similarly, in this study, ANC of <100/mm3 and lower WBC were associated with bacteremia. 

Several other factors have been reported to have an association with bacteremia in children with FN. Children younger than 5 years old and with primary disease diagnoses such as ALL/Burkitt lymphoma, induction phase of ALL, and primary progressive or relapsed disease with bone marrow involvement were more prone to septic complications [10,26,31]. However, no relationship was observed with blood culture positivity in this study. Also, although we did not find an association, prolonged neutropenia, body temperature over 39 °C, recent chemotherapy (≤7 days), and presence of any focal infection were noted to be predictors of bacteremia, as well as acute phase reactants [5,11,26,29]. Previous treatment and prophylactic use of antibiotics are important risk factors for developing serious bacteremia during neutropenia [32]. Although more than half of the studied children were using antibiotics at the time of FN diagnosis, it was not associated with bacteremia. 

While gram-negative pathogens were formerly predominant, increased use of indwelling catheters, quinolone prophylaxis, and broad-spectrum empirical antibacterial therapy led to an increase in the incidence of infections with gram-positive pathogens in cancer patients during the last decades [2]. CNS account for the majority of blood stream infections in children undergoing therapy for malignancy [33,34]. Similarly, in this study, the most common bacterial etiologic agents isolated from blood cultures were CNS. Above all, for a general appropriate approach to infectious complications in neutropenic cancer patients, it is required to obtain knowledge of the local epidemiology of infections, the causative organisms, and their resistance phenotype.

The retrospective design and indigenous disadvantages of blood culture are limitations of this study. Molecular techniques, such as polymerase chain reaction, could have amplified the success of bacterial identification rates, as even the effects of previous antibiotic exposure are omitted. Conventional methods have the ability to yield a microorganism in rates of 40% in children with clinical sepsis and 75% in high-risk febrile neutropenic children [35–37]. 

In conclusion, the association of possible risk factors related to bacteremia in children with FN was investigated in this study. The presence of any bacterial growth in CVC/IP cultures during the previous 3 months and the presence of previous FN episode(s) before the study period were found to be risk factors for bacteremia. We suggest that each patient must be evaluated individually for their own risk factors before the management of FN episodes.
